# Career and life fulfillment and planning for medical trainees, and physicians

**DOI:** 10.5116/ijme.6372.17ba

**Published:** 2022-11-28

**Authors:** Neil J. MacKinnon, Danielle Rosema, Pauwlina Cyca

**Affiliations:** 1 Department of Population Health Sciences, Medical College of Georgia, Augusta University, Augusta, Georgia, USA; 2 Department of Pediatrics, Medical College of Georgia, Augusta University, and the Childrens Hospital of Georgia, Augusta, Georgia, USA; 3 Cumming School of Medicine, University of Calgary, Calgary, Alberta, Canada

**Keywords:** Career and life fulfillment, planning for medical trainees, physicians

## Introduction

In recent years, there has been much attention focused on issues related to burnout, emotional exhaustion, and work-life balance in medical trainees and physicians. A national survey of residents, fellows, and interventional radiologists revealed that 54.3% were at high risk of depersonalization, 61.9% were at high risk of emotional exhaustion, and 71.9% experienced burnout, which the authors conclude has a negative impact on career longevity and satisfaction, mental health, and patient care.^1^ Burnout is often discussed in terms of workplace conflicts, and while the 2019 sentinel report by the National Academies of Sciences, Engineering, and Medicine identified clinician burnout as a major problem, it also recognized that the problem is complex. A chapter in the report was dedicated to student and trainee burnout and professional well-being, and work-home conflict was acknowledged as a contributor to burnout.^2^ Similarly, the authors of a Canadian survey of medical students, residents, and physician teachers in Saskatchewan found high levels of job, career, and parenting dissatisfaction, and recommended that medical schools should offer programs that recognize the family needs of students, trainees, and faculty.^3^ These and other studies demonstrate that we cannot entirely address clinician burnout, dissatisfaction, and exhaustion by only considering workplace factors.

If personal as well as professional factors contribute to burnout, then aligning these areas of life is essential to helping medical trainees reach their potential and become fulfilled. Brown and Gunderman argue that fulfillment is a preferred term over satisfaction, as the former implies the thorough realization of potential.^4^ The interplay between personal and professional factors is evident in a survey of 5210 general pediatricians who took the General Pediatrics Certifying Examination. In this survey, the most important factor in the choice of the first position upon completion of training was lifestyle and spousal or family considerations, cited by 69% of females and 55% of males.^5^ White, in writing about the integration between personal life and healthcare careers, proposes that “Balancing your career with your personal life requires being candid with yourself about your priorities and deciding how you use your time and energy – in other words, optimizing your life choices. Integrating your personal life and career is not just a challenge for women or for dual-career couples with children”.^6^

It is clear, then, that a holistic approach to career and life planning in medicine is needed. We recommend adopting a five-step plan that focuses on holism through a combination of internal reflection and external consultation. This process was developed by one of us (NM) in response to a lack of existing methods in the literature and it fits medical trainees and physicians well as it can be employed at the start of medical training or later on in one’s career. The steps are: (1) scan the healthcare environment, including speaking with mentors, analyzing health workforce data, and reviewing the literature on career fulfillment for one’s chosen specialty or subspecialty; (2) engage in self-reflection and self-assessment, including consideration of one’s definition of success, values, needs, passions, interests, strengths, and abilities; (3) set an overarching career vision with short- and long-term professional and personal goals; (4) formulate an action plan with strategies, a timeframe, and resources needed to accomplish these goals; and (5) partake in regular, ongoing reassessment and reevaluation, accounting for the fact that career and life planning is an iterative process.^7^ When thoughtfully followed, this process can prospectively address clinician burnout in both the short and long term.

This five-step model is a blend of reflection and action and, in order to truly make a difference, should include the action plan (step four). An action plan that achieves life-work balance must include the following: career, community and citizenship, discretionary time and hobbies, faith, finances, health, and relationships. A trusted mentor, advisor, or coach should be consulted when developing this holistic professional and personal plan. Goals will change with age and time and the importance of the seven components may wax and wane with life phases.

If this model is critical to supporting medical to trainees develop career and life plans that will fulfill them, then medical programs have an obligation to revise curricula. This should be done so that students are exposed to the research on career and life planning, and they should be given time to develop their own plans using effective models like the one proposed here, and given tools to apply these plans in an integrated way throughout their education and careers. The topic should be included into the curricula of all medical schools, which would help medical students make informed, evidence-based decisions about career paths, using the peer-reviewed literature on career fulfillment and work-life balance. Once the components of career fulfillment are delineated by a medical student, and barriers or challenges are identified, faculty and staff should assist in the remediation of those challenges and barriers in collaboration with the student and his or her goals.

Residency programs and fellowships should incorporate career fulfillment in the match process and residency program directors should spend time with each resident to develop a career and life plan. There should be focused discussions in these programs that use the existing literature on predictors of career fulfillment in the specialty/sub-specialty. While there are requirements by the Accreditation Council for Graduate Medical Education in the United States related to well-being in residency programs, and the implementation of duty hour restrictions and strategies in scheduling to prioritize fatigue mitigation have helped, more can be done. Mentors can play a critical role in the lives of residents and should be checking in on the well-being of residents on a regular basis. If trainees are able to find fulfillment in their day-to-day lives, this will ultimately better the person as a whole, with the downstream effect of providing better care to patients.

Beyond medical school, practicing physicians should be given opportunities to engage in continuing education related to career and life planning. Continuing education is important because the experiences, interests, and life circumstances of physicians change over time. Thus, including this topic in continuing medical education events would be worthwhile. Moreover, practicing physicians should reflect upon, and revise, as necessary, their career and life plan on a regular basis. We recommend doing this every six months and doing it with a mentor, trusted colleague or life coach.

Through our perspectives of a medical student, a recent resident, and faculty member at a college of medicine, we have provided an approach to overcome this education and training deficit. The ultimate goal of addressing this gap is to align career and personal planning, which will increase the chances of personal and professional fulfillment. The consequences of inaction are significant, as has been noted by the National Academies of Sciences, Engineering, and Medicine. We agree with advice that has been provided elsewhere, “We ignore the subject of physician fulfillment at our peril. For academic medicine to thrive in the coming years, we need to attend more carefully than ever to the factors that enhance and detract from the quality of work we do.”^4^

In summary, as we have argued, while clinician burnout, dissatisfaction, and exhaustion are well-documented and common issues, we cannot address them by only considering workplace factors. Since both professional and personal factors contribute to burnout, alignment of these factors can positively influence career and life fulfillment. A five-step process can be used to ensure alignment and this plan should include seven essential components of a career and life plan: career, community and citizenship, discretionary time and hobbies, faith, finances, health, and relationships. We have proposed an approach to integrate this into medical education, residency training, and professional development for practicing physicians. With an aging physician workforce in many Western countries, along with a shortage of physicians, the cost of inaction of career and life planning in medical students and other trainees will be significant.

### Acknowledgements

The input of Dr Candis Bond of Augusta University is greatly appreciated.

### Conflict of Interest

The authors declare that they have no conflict of interest.
